# Effects of Ocean Acidification on Molting, Oxidative Stress, and Gut Microbiota in Juvenile Horseshoe Crab *Tachypleus tridentatus*

**DOI:** 10.3389/fphys.2021.813582

**Published:** 2022-01-06

**Authors:** Ximei Liu, Jiani Liu, Kai Xiong, Caoqi Zhang, James Kar-Hei Fang, Jie Song, Zongguang Tai, Quangang Zhu, Menghong Hu, Youji Wang

**Affiliations:** ^1^Key Laboratory of Exploration and Utilization of Aquatic Genetic Resources, Ministry of Education, Shanghai Ocean University, Shanghai, China; ^2^Shanghai Engineering Research Center of Aquaculture, Shanghai Ocean University, Shanghai, China; ^3^Department of Applied Biology and Chemical Technology, The Hong Kong Polytechnic University, Kowloon, Hong Kong SAR, China; ^4^Tianjin Era Biology Technology Co., Ltd., Tianjin, China; ^5^Shanghai Skin Disease Hospital, Tongji University School of Medicine, Shanghai, China

**Keywords:** ocean acidification, horseshoe crab, molting, ecdysone, oxidative stress, gut microbiota

## Abstract

Anthropogenic elevation of atmospheric carbon dioxide (CO_2_) drives global-scale ocean acidification (OA), which has aroused widespread concern for marine ecosystem health. The tri-spine horseshoe crab (HSC) *Tachypleus tridentatus* has been facing the threat of population depletion for decades, and the effects of OA on the physiology and microbiology of its early life stage are unclear. In this study, the 1st instar HSC larvae were exposed to acidified seawater (pH 7.3, pH 8.1 as control) for 28 days to determine the effects of OA on their growth, molting, oxidative stress, and gut microbiota. Results showed that there were no significant differences in growth index and molting rate between OA group and control group, but the chitinase activity, β-NAGase activity, and ecdysone content in OA group were significantly lower than those of the control group. Compared to the control group, reactive oxygen species (ROS) and malondialdehyde (MDA) contents in OA group were significantly increased at the end of the experiment. Superoxide dismutase (SOD), catalase (CAT), and alkaline phosphatase (AKP) activities increased first and then decreased, glutathione peroxidase (GPX) decreased first and then increased, and GST activity changed little during the experiment. According to the result of 16S rRNA sequencing of gut microbiota, microbial-mediated functions predicted by PICRUSt showed that “Hematopoietic cell lineage,” “Endocytosis,” “*Staphylococcus aureus* infection,” and “Shigellosis” pathways significantly increased in OA group. The above results indicate that OA had no significant effect on growth index and molting rate but interfered with the activity of chitinolytic enzymes and ecdysone expression of juvenile horseshoe crabs, and caused oxidative stress. In addition, OA had adverse effects on the immune defense function and intestinal health. The present study reveals the potential threat of OA to *T. tridentatus* population and lays a foundation for the further study of the physiological adaptation mechanism of juvenile horseshoe crabs to environmental change.

## Introduction

The ongoing increase of anthropogenic carbon dioxide (CO_2_) in the atmosphere due to human fossil fuel combustion and deforestation causes an accumulation of CO_2_ in the oceans. As much as one-third of anthropogenic CO_2_ is absorbed by the oceans, ocean acidification (OA) has been frequently found worldwide (Doney et al., 2009). Surface ocean pH is already 0.1 unit lower than preindustrial values and will continue to decrease by 0.3–0.4 pH units by the end of the 21st century (Orr et al., 2005). It is predicted that the maximum pH reduction at the ocean surface will reach 0.77 units by 2,300 (Fabry et al., 2008). This global-scale OA driven by the excessive emission of anthropogenic CO_2_ is changing the hydrochemical environment on which marine organisms depend. Previous studies showed that OA usually has a negative or neutral effect on growth, survival (Long et al., 2013), reproduction (Kroeker et al., 2010), physiology (Qyli et al., 2020), and feeding behavior (Dodd et al., 2015; McLuckie et al., 2021) of studied marine organisms, especially the calcified organisms, such as mollusks, corals, and crustaceans (Long et al., 2013), because OA can reduce the saturation of carbonate minerals, increase the dissolution rate of calcium carbonate, and result in the development obstruction of marine calcifiers, the decline of calcification rate, and physiological stress. It may also reduce the biodiversity, leading to the extinction of some key species in the food chain, thus affecting the interaction between species and ultimately affecting the stability of marine ecosystems (Rossoll et al., 2012; Gaylord et al., 2015).

Horseshoe crabs are regarded as marine living fossils and have lived on the earth for nearly 500 million years (Rudkin and Young, 2009). They are ecologically important as their eggs serve as a major protein source for migratory shorebirds, gulls, and fish (Botton and Shuster, 2003). During the reproductive season, female adult horseshoe crabs lay their eggs in the sands of the high tide zone. Juvenile horseshoe crabs mostly live in the intertidal mudflats, with a pH range of 6.99–8.62 (measured at estuarine waters inundated the intertidal area) in the habitats, where pH fluctuation or OA frequently occurs (Meilana et al., 2021). Chen et al. (2004) referred horseshoe crabs as a good indicator species for reflecting the general health of the coastal habitat. Unfortunately, habitat destruction and overfishing have caused a sharp decline in the horseshoe crab population over the last 30 years (Lee and Morton, 2016; Fu et al., 2019; Liao et al., 2019). The horseshoe crab resources in China have already faced exhaustion. In 2019, *Tachypleus tridentatus* was listed as “endangered” in IUCN Red List (Laurie et al., 2019). The revised “List of key protected wild animals in China” in 2021 officially listed tri-spine horseshoe crab as the second-class national protected wild animal. Current studies on horseshoe crab mainly focus on population survey (Xie et al., 2019), resource conservation (Zhu et al., 2020), artificial breeding (Tinker-Kulberg et al., 2020), blood collection (John et al., 2011; Owings et al., 2019), and recombinant Tachyplesin (Yu et al., 2020). The impact of environmental factors on horseshoe crabs is mainly concentrated on heavy metals, salinity, temperature, and sediments (Billy et al., 2015). Horseshoe crabs, as an arthropod, need to experience 15–16 times of molting stages before they eventually develop into adults, which takes more than 10 years (Hong, 2011). Studies have shown that individual organisms at the larval stage are sensitive to changes in the external environment, and elevated CO_2_ can damage their growth (Giltz and Taylor, 2017) and reduce the survival rate of some arthropods (Long et al., 2013). Therefore, understanding the effects of OA on the growth of juvenile horseshoe crab is essential to predict the future changes in the population of tri-spine horseshoe crabs. However, the impacts of OA on growth, physiology, and intestinal flora of horseshoe crabs are lacking, which need to be clarified for better protecting horseshoe crabs.

In the present study, juvenile horseshoe crabs were exposed to acidified seawater (pH 7.3) for 28 days to explore the potential impact of continuous OA on juvenile horseshoe crabs in terms of growth, molting, oxidative stress, and intestinal microbiome changes, and reveal the possible threat of OA to tri-spine horseshoe crab population. This study can not only lay a foundation for the study of the physiological adaptation mechanism of the juvenile horseshoe crab to OA but also accumulate basic data for the assessment of the impact of climate change on the marine ecosystem.

## Materials and Methods

### Experimental Animals and Conditions

Artificially bred juvenile horseshoe crabs were collected from Beihai, Guangxi province, China, and transported to the shellfish laboratory of Shanghai Ocean University. Prior to the experiment, 720 healthy and vigorous 1st instar larvae (initial wet weight: 0.0208 ± 0.0015 g, precursor width: 5.69 ± 0.42 mm) were randomly selected from the circulating aquaculture system and divided into six glass tanks (30 cm × 20 cm × 15 cm), and the bottoms of these tanks were covered with 2 cm-thick fine sand. Juvenile horseshoe crabs were kept for 2 weeks under the following environmental conditions: water temperature 25–26°C, salinity 28–31‰, pH 8.1–8.2, dissolved oxygen 6–8 mg/L, and photoperiod 12 h (natural light 6:00–18:00). Artificial seawater in each tank was renewed once daily (18:00) with 1/2 of the total volume. The 1st instar larvae mainly rely on their own yolk nutrients to maintain basic metabolism, thus no feeding was provided (Hong, 2011).

After 2 weeks of acclimation, two levels of seawater pH were designed. Three glass tanks of juvenile horseshoe crabs were randomly selected as the acidification treatment [pH 7.3, Meilana et al. (2021) found that estuarine waters inundated the intertidal area with pH fluctuation of 6.99–8.62, and pH 7.3 is the extreme acidified pH expected to be reached by 2300 in the ocean (Caldeira and Wickett, 2005)], and the other three tanks of juvenile horseshoe crabs were set as the control group (pH 8.1 is the normal environmental pH and the normal artificial seawater is also pH 8.1 in the laboratory). Pure CO_2_ gas was filled into the seawater to achieve real-time pH control and monitoring via a *p*CO_2_/pH system equipped with a pH meter (WTW 3310) and pH electrode (SenTix 41) designed by Loligo Systems Inc. (Shang et al., 2018; Wu et al., 2018). Within 3 h, the pH gradually decreased from 8.1 to 7.3. Seawater salinity was measured by using a saline concentration refractometer (MASTER-S28M, Japan). Titration was used to determine the total alkalinity (TA). The carbonate chemical parameters such as dissolved inorganic carbon (DIC), *p*CO_2_, calcite saturation (Ωcal), and aragonite saturation (Ωara) in experimental water were calculated by CO_2_SYS (Lewis et al., 2013). During the experiment, seawater was renewed with the same pH value that was aerated in advance, and the other environmental conditions were consistent with the acclimation period.

### Sample Collection

On the 0, 7th, 14th, and 28th days of the experimental exposure, three samples of each parallel tank at each time point were collected to determine reactive oxygen species production immediately. In addition, 12 samples were randomly collected from each parallel tank at each time point and placed in a −80°C refrigerator for subsequent determination of molting-related enzymes, hormones, and antioxidant enzymes. On the 28th day, the juvenile horseshoe crab intestines were taken out under an anatomical microscope and the specific operation steps are as follows: first, the juvenile horseshoe crab was dried with absorbent paper, and placed in a sterile petri dish with ventral surface up. Then under the anatomical microscope, one forceps fixed the horseshoe crab, another forceps carefully removed the appendages. Lastly, the cephalothorax and opisthosoma were gently pulled, at which point the intestine was pulled out and put in a 2 mL RNase-free sterile centrifuge tube. Then intestinal samples were quickly frozen with liquid nitrogen and stored at a −80°C refrigerator for subsequent intestinal microbial analysis.

### Determination of Chitinolytic Enzymes

Chitinase and *N*-acety-β-D-glucosidase (β-NAGase) activity were measured using the kits produced by Nanjing Jiancheng Bioengineering Research Institute (Nanjing, China), and all parameters of enzyme activity and reactive oxygen species (ROS) below were determined by a microplate reader (Synergy H4, BioTek, United States).

#### Chitinase

The collected juvenile horseshoe crabs were homogenized in an ice bath according to the ratio of tissue mass (g): volume of extracted liquid (mL) of 1:10, and then centrifuged at 10,000 *g* for 20 min at 4°C. The supernatant was taken and placed on ice for testing. Chitinase hydrolyzes chitin to produce *N*-acetylglucosamine, which is further combined with 3,5-dinitrosalicylic acid to produce a brownish-red compound with a characteristic absorption peak at 540 nm. One chitinase activity unit (U) is the amount of enzyme that decomposed chitin to produce 1 mol *N*-acetylglucosaminidase by 1 g of tissue per hour at 37°C.

#### β-NAGase

Samples were mechanically homogenized in an ice bath according to the ratio of tissue weight (g): volume of physiological saline (mL) = 1:9, and then centrifuged at 600 g for 10 min. The supernatant was diluted with 0.9% physiological saline to prepare a 1% homogenate for testing.

*P*-nitrobenzene glucoside was used as the reaction substrate, and the substrate was hydrolyzed under the action of β-NAGase to release free *p*-nitrophenol. The reaction was carried out at 37°C for 15 min, the alkaline solution was added to stop the reaction, and the absorbance was measured at 400 nm. At 37°C, 1 g tissue protein reacted with the substrate for 1 min and hydrolyzed to produce 1 mol *p*-nitrophenol was calculated as one unit of β-NAGase.

### Ecdysone

The samples were washed with pre-cooled PBS (pH = 7.4). The tissues were cut into pieces after weighing and adding PBS according to the ratio of tissue weight (g): volume (mL) = 1:9. Tissues were fully homogenized on ice by an ultrasonic homogenizer. Finally, the homogenate was centrifuged at 5,000 *g* for 10 min, and the supernatant was taken for testing. The concentration of ecdysone in juvenile horseshoe crab was determined according to the instruction of Ecdysone Elisa kit (Shanghai Zhuocai Biotechnology Co., Ltd.).

### Reactive Oxygen Species

2,7-Dichlorodi-hydrofluorescein diacetate (DCFH-DA) was used as a ROS-specific fluorescent dye to analyze the ROS content. According to the instructions of the ROS assay kit (Nanjing Jiancheng Bioengineering Institute, Nanjing, China), the tissue was cut into pieces, and the single-cell suspension was prepared by the enzyme digestion method. Then DCFH-DA fluorescent probe (10 μmol/L) was added. Finally, fluorescence intensity was detected with excitation and emission peaks of 488 and 528 nm. The ROS content in tissue cells was expressed by the DCF fluorescence intensity.

### Antioxidant and Non-specific Immune Enzyme Activity

Superoxide dismutase (SOD), catalase (CAT), malondialdehyde (MDA), glutathione peroxidase (GPX), glutathione-*S*-transferase (GST), and alkaline phosphatase (AKP) were measured using the assay kits produced by Nanjing Jiancheng Bioengineering Research Institute. The SOD activity is determined by the xanthine oxidase method (Elstner and Heupel, 1976). CAT activity was determined according to the method of ammonium molybdate method (Góth, 1991). The MDA content was assessed by the thiobarbituric reactive species (TBARS) assay, which represented the level of lipid peroxidation (Ohkawa et al., 1979). AKP activity was measured based on the method of King (1965). GPX activity was measured according to the method of Lawrence and Burk (1976), GSH activity was measured by the method of Ringwood et al. (1999). GST activity was measured according to the method of Habig et al. (1974).

### Intestinal Microbiota Analysis

Ten juvenile horseshoe crab intestines were mixed as one sample in each tank, and each group had three replicates. Samples were sent to Shanghai Oe Biotech. Co., Ltd. (Shanghai, China) for sequencing with MiSeq (Illumina, San Diego, CA, United States). Based on 16S rRNA high-throughput sequencing technology, primers were designed in the V3–V4 region for PCR amplification. Subsequently, the obtained original data were de-interleaved and spliced. Clean reads were subjected to primer sequences removal and clustering to generate operational taxonomic units (OTUs) using UCHIME (version 2.4.2) software according to 97% similarity cutoff. RDP classifier was used to annotate representative sequences and blasted against Silva database (Version 132) to obtain annotation information of OTU (confidence threshold was 70%). On this basis, alpha diversity and community abundance were further analyzed.

### Statistical Analysis

All data were expressed as mean ± standard deviation (SD). IBM SPSS Statistics 26 software was used for statistical analysis. Prior to the analysis, the normality of the data was evaluated by using the Shapiro–Wilk’s test, and homogeneity of variances was checked using Levene’s test. Two-way ANOVA was used to compare the effects of time and pH interaction on juvenile horseshoe crab indexes. If there was a significant interaction between the two factors, *t*-test was used to analyze the effects of different pH on juvenile horseshoe crab indexes at each sampling time, and one-way ANOVA was used to detect the difference of each index at different times. Biplot of principal component analysis (PCA) was performed on the physiological parameters by using XLSTAT^®^ 2014. To understand the covariation trend among variables, correlations between variables were calculated according to the Pearson coefficient (*r*), and only the correlations with | *r*| > 0.4 and *P* < 0.05 were considered. Alpha diversity, including Chao1 index, Shannon index, and Simpson index, was used to estimate the microbial diversity of intestinal samples. Weighted Unifrac principal coordinate analysis (PCoA) was performed using the Unifrac distance matrix calculated by QIIME software. PICRUSt functional prediction analysis is based on 16S sequencing data annotated by the Greengenes database to predict the functional composition of known microbial genes to count the functional differences between different groups.

## Results

### Seawater Chemistry Parameters

The carbonate chemistry parameters of seawater within each treatment are summarized in Table 1. During the 28 days of experiment, the water temperature was maintained at 25.5 ± 0.5°C, and the salinity value was maintained at 29.5 ± 1.3 psu during the experiment.

**TABLE 1:** The composition of seawater carbonate chemicals, temperature (°C), salinity (psu), pH, total alkalinity (TA; μmol kg^–1^), dissolved inorganic carbon (DIC, μmol kg^–1^), *p*CO_2_ (μatm), saturation state for calcite (Ωcal), and aragonite (Ωara) in this experiment were summarized in the table (mean ± SD, *n* = 3).

Groups	Temperature (°C)	Salinity (psu)	pH	TA (μmol kg^–1^)	DIC (μmol kg^–1^)	*p*CO_2_ (μatm)	Ωcal	Ωara
pH 8.1	25.5 ± 0.45	29.5 ± 1.22	8.10 ± 0.03	2325 ± 22	2033 ± 22	362 ± 22	4.85 ± 0.25	2.92 ± 0.04
pH 7.3	25.6 ± 0.42	29.4 ± 1.25	7.35 ± 0.04	2287 ± 25	2328 ± 16	2658 ± 89	0.88 ± 0.06	0.47 ± 0.02

### Molting Rate

**FIGURE 1:**
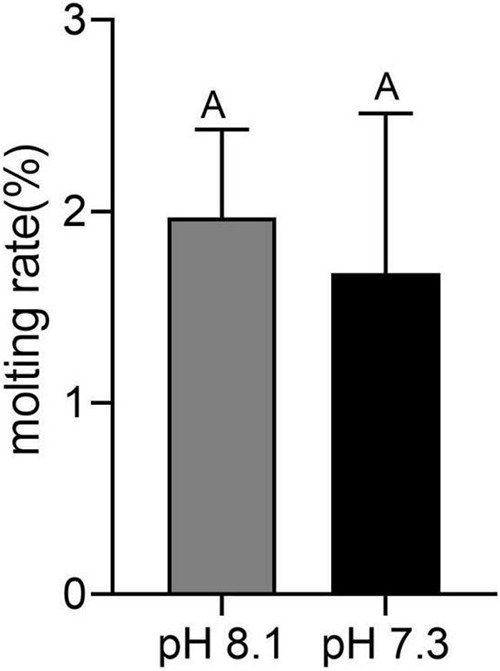
Molting rate of juvenile horseshoe crabs after 28 days’ acidification exposure. Capital letters represent significant difference between two groups (*P* < 0.05).

During the whole experiment, no juvenile *T. tridentatus* died in both the control group and the acidification group. At the end of the experiment, the wet weight of the control group was 0.0205 ± 0.0001 g, and the width of the precursor was 5.63 ± 0.36 mm. The wet weight of the acidification group was 0.0207 ± 0.0013 g, and the width of the precursor was 5.42 ± 0.26 mm. There was no significant difference in growth index between the control group and the acidification group (*P* > 0.05). Molting occurred in six juvenile horseshoe crabs in the acidification group, with a molt rate of 1.7%, and seven juvenile horseshoe crabs in the control group, with a molting rate of 1.9%. There was no significant difference in the molting rate between the two groups (Figure 1, *P* > 0.05).

### Chitinase and β-NAGase Activity

**FIGURE 2:**
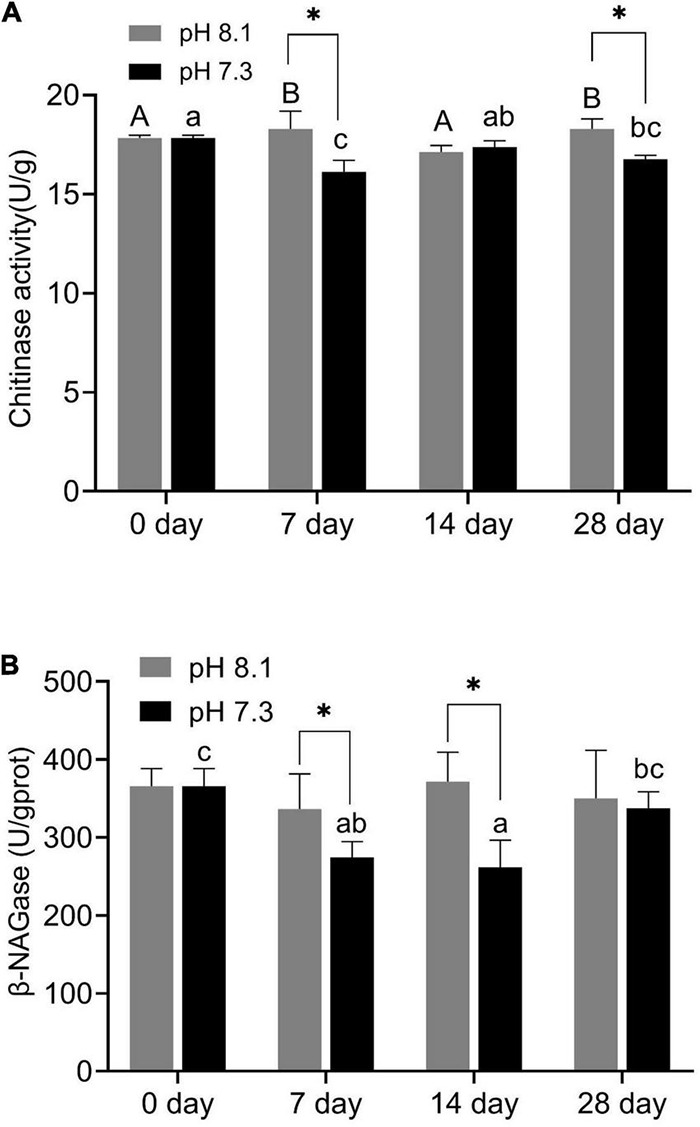
Chitinase activity **(A)** and β-NAGase activity **(B)** of juvenile horseshoe crabs after 28 days’ acidification exposure. Asterisks indicate significant difference between two pH levels (*P* < 0.05). Different capital letters and different lowercase letters represent significant difference in pH 8.1 and pH 7.3 at different sampling times (*P* < 0.05).

During the experiment, pH and the interaction between pH and time had significant effects on chitinase activity (*P* < 0.05, Table 2). On the 7th and 28th days of the experiment, the chitinase activity in the acidification group was significantly lower than that in the control group (*P* < 0.05, Figure 2A). On the 14th day, there was no significant difference between the two groups (Figure 2A).

**TABLE 2:** Summary of two-way ANOVA results on effects of pH and time on the physiological parameters of *T. tridentatus*.

Factor	Degree of freedom	Mean square	*F*	*P*
Chitinase				
Time	3	0.489	2.328	0.113
pH	1	4.491	21.370	0.000
Time*pH	3	2.091	9.951	0.001
β-NAGase				
Time	3	4436.416	3.456	0.042
pH	1	12084.688	9.975	0.006
Time*pH	3	3788.470	2.951	0.064
Ecdysone				
Time	3	42.825	4.960	0.013
pH	1	183.121	21.211	0.000
Time*pH	3	42.959	4.976	0.013
ROS				
Time	3	4811.611	7.690	0.002
pH	1	7350.000	11.747	0.003
Time*pH	3	1894.556	3.028	0.060
MDA				
Time	3	0.057	7.819	0.002
pH	1	0.109	15.042	0.001
Time*pH	3	0.025	3.486	0.041
CAT				
Time	3	140.331	5.243	0.010
pH	1	21.856	0.817	0.380
Time*pH	3	98.12	3.66	0.035
SOD				
Time	3	1113.096	5.175	0.011
pH	1	2009.271	9.341	0.008
Time*pH	3	587.166	2.730	0.078
GST				
Time	3	113.844	4.318	0.021
pH	1	0.169	0.006	0.937
Time*pH	3	3.769	0.143	0.933
GPX				
Time	3	557.388	7.472	0.002
pH	1	24.522	0.329	0.574
Time*pH	3	1159.403	15.541	0.000
AKP				
Time	3	11.056	8.416	0.001
pH	1	48.269	36.741	0.000
Time*pH	3	11.577	8.812	0.001

*β-NAGase, N-acetyl-β-D-glucosaminidase; ROS, reactive oxygen species; SOD, superoxide dismutase; CAT, catalase; MDA, malondialdehyde; AKP, alkaline phosphatase; GST, glutathione S-transferase; GPX, glutathione peroxidase; pH (pH 7.3, pH 8.1), time (0, 7th, 14th, and 28th days).*

β-NAGase activity was significantly affected by pH and time (*P* < 0.05, Table 2). On the 7th and 14th day of the experiment, β-NAGase activity of the acidification group was significantly lower than that of the control group. While on the 28th day, the β-NAGase activity had no significant difference between the two groups. The β-NAGase activity of the acidification group first decreased and then increased on the whole (Figure 2B).

### Ecdysone

**FIGURE 3:**
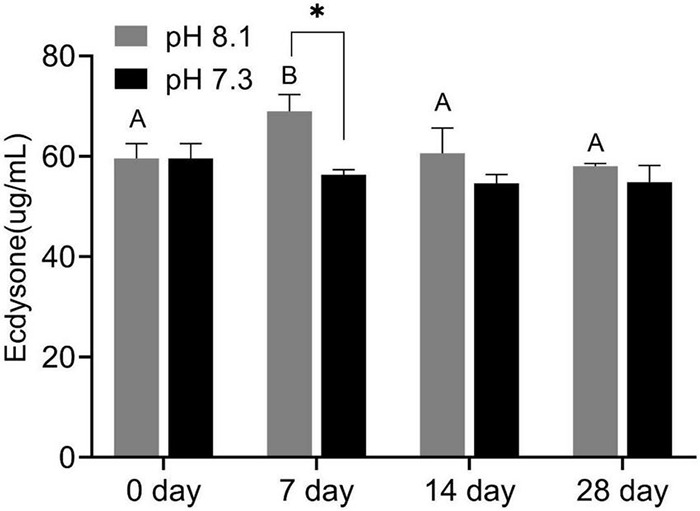
Ecdysone content of juvenile horseshoe crabs after 28 days’ acidification exposure. Asterisks indicate significant difference between two pH levels (*p* < 0.05). Different capital letters represent significant difference at different sampling times (*P* < 0.05).

During the experiment, ecdysone content was significantly affected by time, pH, and their interaction (*P* < 0.05, Table 2). On the 7th day, the ecdysone concentration in the acidification group was significantly lower than that in the control group (*P* < 0.05). On the 14th and 28th days, ecdysone content had no significant difference between the two groups (*P* > 0.05, Figure 3).

### Reactive Oxygen Species and Malondialdehyde

**FIGURE 4:**
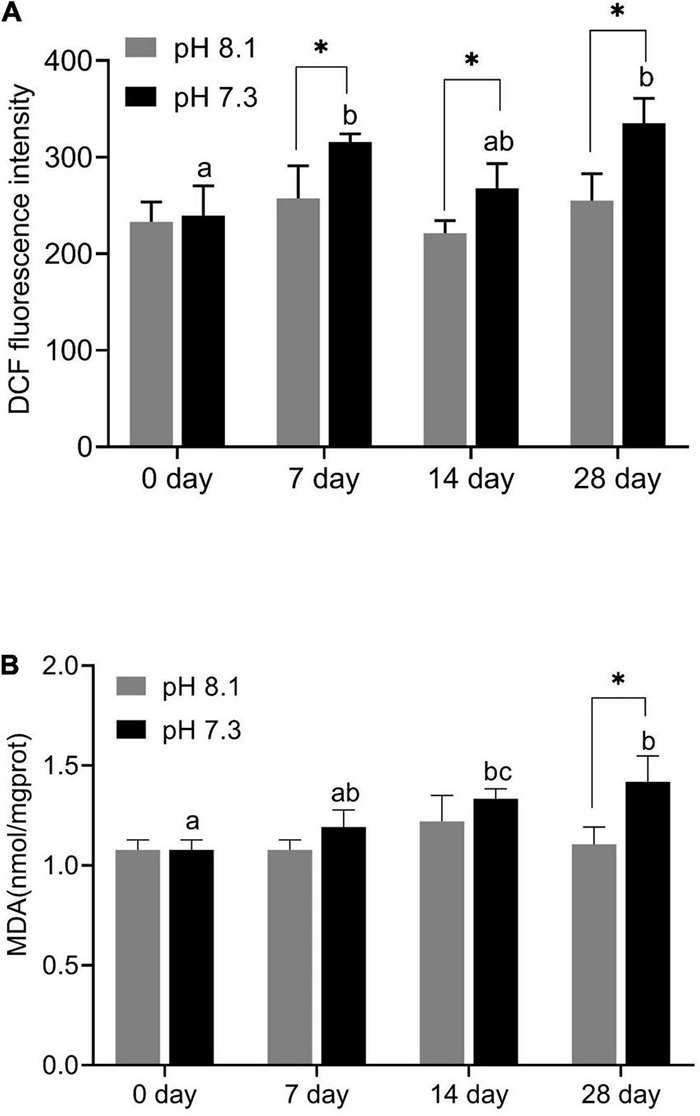
Reactive oxygen species (ROS) content **(A)** and MDA content **(B)** of juvenile horseshoe crabs after 28 days’ acidification exposure. Asterisks indicate significant difference between two pH levels (*P* < 0.05). Different lowercase letters represent significant difference at different sampling times (*P* < 0.05).

During the experiment, ROS content was significantly affected by pH and time (*P* < 0.05), but their interaction had no significant effect on ROS content (Table 2). Acidification conditions significantly increased the production of ROS. During the whole experimental period, the content of ROS in the acidification group was significantly higher than that in the control group (*P* < 0.05, Figure 4A).

During the experiment, MDA content was significantly affected by time, pH, and their interaction (*P* < 0.05, Table 2). On the 7th and 14th days, MDA content had no significant difference between the two groups. With the extension of acidification exposure, the MDA content in the acidification group gradually increased. On the 28th day, MDA content in the acidification group was significantly higher than that in the control group (*P* < 0.05, Figure 4B).

### Antioxidant Enzymes and Alkaline Phosphatase

During the experiment, CAT and GPX activities were significantly affected by pH and the interaction between pH and time (*P* < 0.05); SOD was significantly affected by pH and time (*P* < 0.05), but the interaction between pH and time had no significant effect on SOD (*P* > 0.05); time has a significant effect on GST, while the interaction between pH and time had no significant effect on GST; AKP activity was affected by time, pH, and their interaction (*P* < 0.05, Table 2).

**FIGURE 5:**
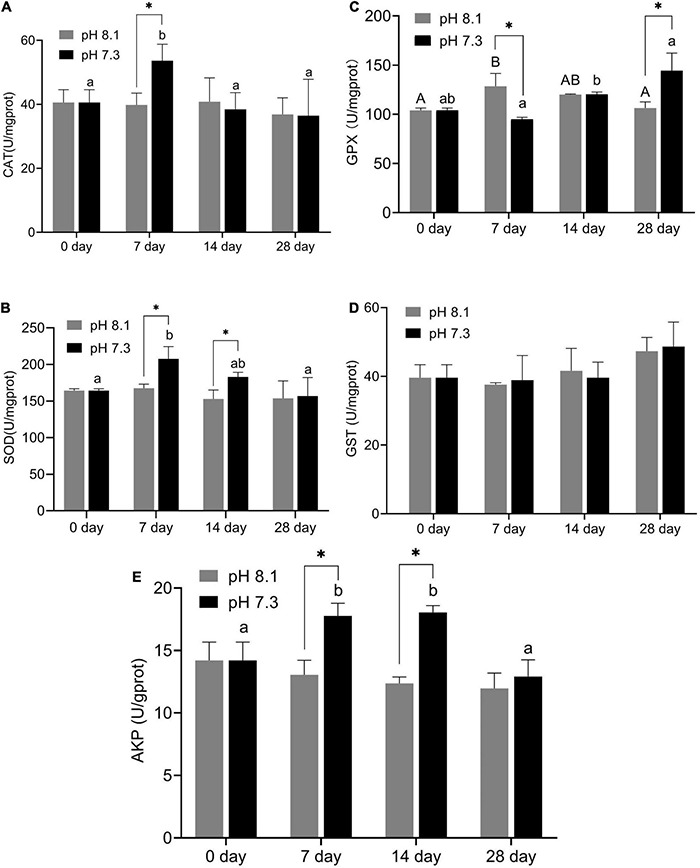
Catalase (CAT) activity **(A)**, SOD activity **(B)**, GPX activity **(C)**, GST activity **(D)**, and AKP activity **(E)** of juvenile horseshoe crabs after 28 days’ acidification exposure. Asterisks indicate significant difference between two pH levels (*P* < 0.05). Different capital letters and different lowercase letters represent significant difference in pH 8.1 and pH 7.3 at different sampling times (*P* < 0.05).

On the 7th day, CAT activity significantly increased (*P* < 0.05) and returned to normal levels on the 14th and 28th days (Figure 5A); SOD activity in the acidification group was significantly higher than that in the control group on the 7th and 14th days (*P* < 0.05, Figure 5B); GPX activity in the acidification group was significantly lower than that in the control group on the 7th day, then slowly increased, and was significantly higher than that in the control group on the 28th day (*P* < 0.05, Figure 5C); GST activity did not change significantly during the whole experiment period (Figure 5D); AKP activity in the acidification group was significantly higher than that in the control group on the 7th and 14th day (*P* < 0.05, Figure 5E).

### Principal Component Analysis and Correlation Analysis

**FIGURE 6:**
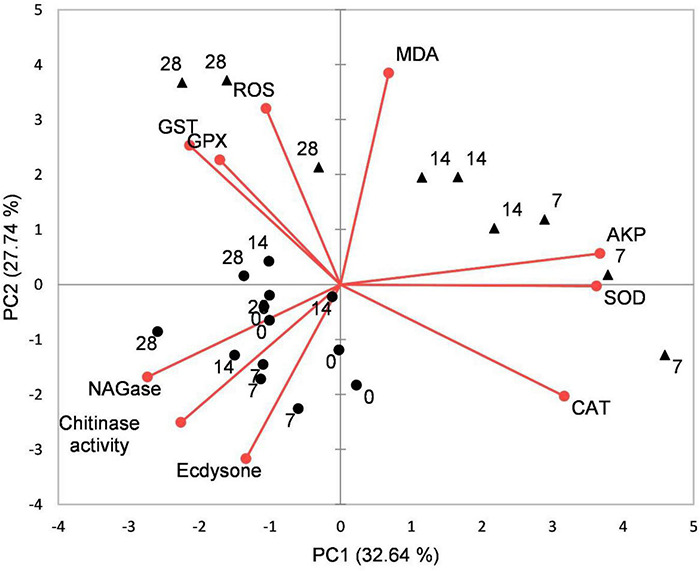
Biplot originating from principal component analysis (PCA) integrating all measured variables (Chitinase, β-NAGase, Ecdysone, ROS, MDA, CAT, SOD, GPX, GST, and AKP) and four sampling times (0, 7th, 14th, and 28th days) at different treatments (•–pH 8.1, ▲–pH 7.3).

Principal component analysis results showed that the effects of acidification and time on the oxidative stress response, chitinase, and ecdysone of juvenile horseshoe crabs account for 60.37% of the total components. PC1 accounts for 32.54% of the total variance. This axis represents the specific response of the acidification treatment, which separated the control group and the acidification treatment group. PC2 accounts for 27.74% of the total variance, describing the specific response of exposure time. The 0 and 28th days are separated from the 7th and 14th days (Figure 6).

Pearson correlation analysis showed that chitinase was significantly positively correlated with ecdysone and negatively correlated with MDA. β-NAGase was significantly negatively correlated with SOD and AKP activities. ROS was significantly positively correlated with GPX and MDA. CAT had a significant positive correlation with SOD and AKP but a significant negative correlation with GST and GPX. MDA was positively correlated with GPX (*P* < 0.05, Table 3).

**TABLE 3:** The Pearson coefficient (*r*) among different parameters of *T. tridentatus*.

		Chitinase	β-NAGase	Ecdysone	ROS	CAT	SOD	GST	GPX	AKP	MDA
Chitinase	*r*	1									
	*P*	/									
β-NAGase	*r*	0.296	1								
	*P*	0.16	/								
Ecdysone	*r*	0.429	0.294	1							
	*P*	**0.037**	0.163	/							
ROS	*r*	−0.168	−0.069	−0.390	1						
	*P*	0.43	0.75	0.06	/						
CAT	*r*	−0.302	−0.096	−0.009	−**0.439**	1					
	*P*	0.152	0.65	0.97	**0.03**	/					
SOD	*r*	−0.401	−**0.559**	−0.118	−0.092	**0.558**	1				
	*P*	0.052	**0.04**	0.58	0.67	**0.004**	/				
GST	*r*	−0.142	0.054	−0.219	0.367	−**0.603**	−0.358	1			
	*P*	0.51	0.80	0.30	0.07	**0.002**	0.085	/			
GPX	*r*	−0.123	0.170	0.065	**0.570**	−**0.462**	−0.226	0.237	1		
	*P*	0.57	0.43	0.66	**0.003**	**0.023**	0.28	0.27	/		
AKP	*r*	−**0.419**	−**0.638**	−0.329	−0.112	**0.462**	**0.646**	−0.371	−0.259	1	
	*P*	**0.04**	**<0.01**	0.12	0.60	**0.023**	**<0.01**	0.075	0.22	/	
MDA	*r*	−**0.548**	−0.304	−**0.546**	**0.604**	−0.165	0.136	0.163	**0.583**	0.128	1
	*P*	**0.006**	0.15	**0.006**	**0.002**	0.44	0.62	0.45	**0.003**	0.55	/

*β-NAGase, N-acetyl-β-D-glucosaminidase; ROS, reactive oxygen species; SOD, superoxide dismutase; CAT, catalase; MDA, malondialdehyde; AKP, alkaline phosphatase; GST, glutathione S-transferase; GPX, glutathione peroxidase.*

### Changes in Intestinal Microbiota

#### Sequencing Data

The data volume of clean tags after quality control is distributed between 58,084 and 65,640. The valid tags were distributed between 40,157 and 50,656, and the average length of valid tags is distributed between 408.7 and 412.79 bp. 4,242 OUTs are generated, and the number of OTUs in each sample is distributed between 1,634 and 2,085. The coverage of the acidification treatment group and the control group was close to 1, indicating that the sequencing depth is sufficient to reflect the composition of the entire intestinal microbe (Table 4).

**TABLE 4:** Statistics of the Alpha index of intestinal microbiome.

	OTU	Chao1	Coverage	Shannon	Simpson
7.3	2032 ± 13.38	2138.62 ± 25.33	0.9947 ± 0.00038	9.06 ± 0.0234	0.9934 ± 0.0002
8.1	1876 ± 131.23	2045.25 ± 95.91	0.9937 ± 0.00139	8.73 ± 0.4858	0.9910 ± 0.0038

#### Alpha Diversity and Community Structure Analysis

**FIGURE 7:**
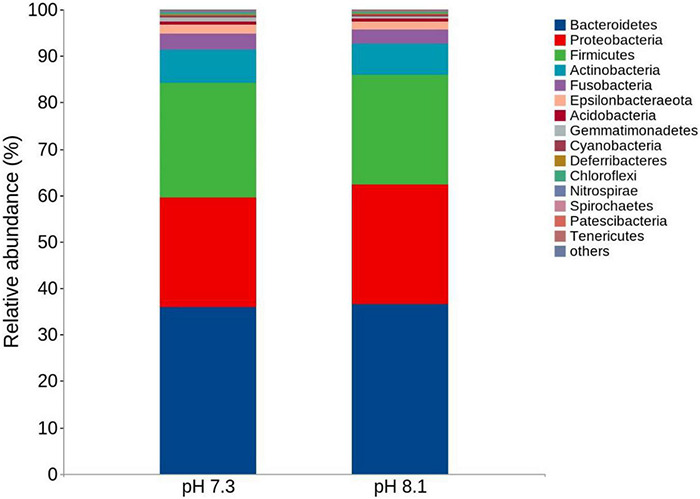
Top 15 relative abundance of the intestinal bacteria in juvenile horseshoe crabs from four different periods at the phylum level.

**FIGURE 8:**
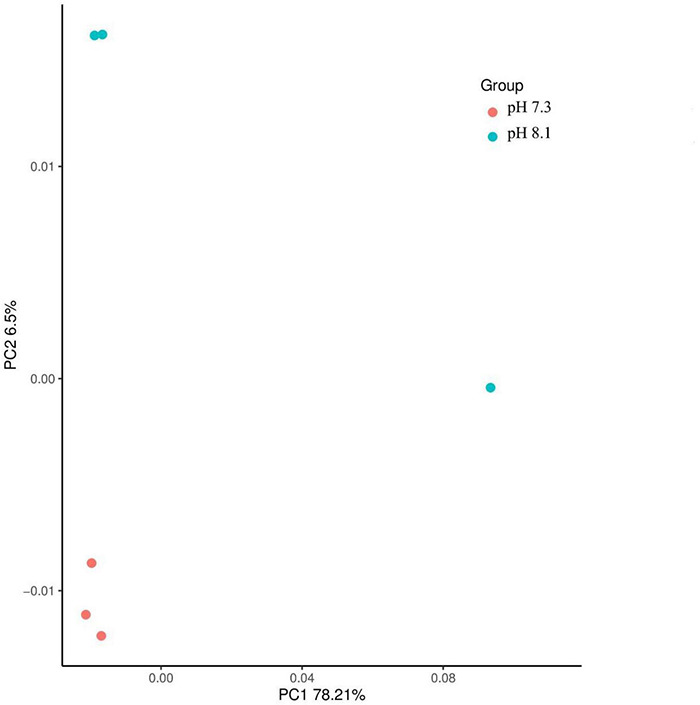
Principal components analysis of juvenile horseshoe crabs intestinal microbiome.

There were no significant differences in terms of diversity index (Shannon and Simpson) and abundance index (Chao1) between the acidification treatment group and the control group (*P* > 0.05, Table 4). At the phylum level, the proportion of five dominant bacteria in the acidification group was Bacteroidetes (35.99%), Proteobacteria (23.48%), Firmicutes (24.82%), Actinobacteria (7.01%), and Fusobacteria (3.60%). The proportion of five dominant bacteria in the control group was Bacteroidetes (36.47%), Proteobacteria (25.75%), Firmicutes (23.67%), Actinobacteria (6.72%), and Fusobacteria (3.12%) (Figure 7). The PCoA analysis based on the Weighted Unifrac distance algorithm showed that PC1 and PC2 explained 84.71% of the differences in the intestinal microbiome (Figure 8).

#### Unique Biomarkers

**FIGURE 9:**
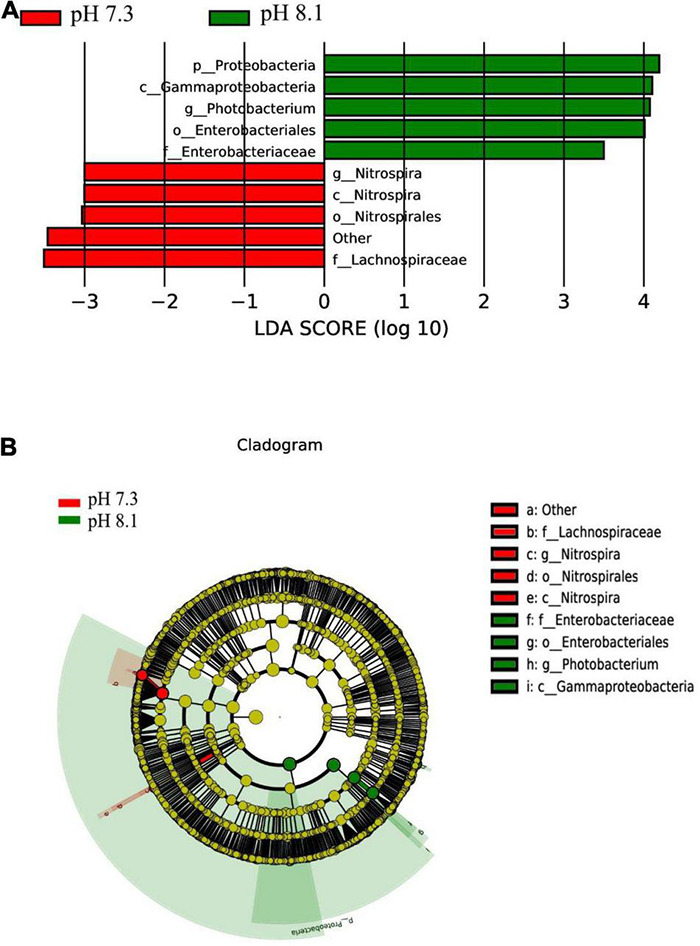
LEfSe analysis of intestinal microbiome biomarker between two pH levels. Bar chart **(A)** showing the LDA scores of bacterial taxa (LDA score > 3, *P* < 0.05), and cladogram **(B)** showing the phylogenetic relationships of bacterial taxa revealed by LEfSe.

The LEfSe analysis between the acidification treatment group and the control group revealed that Proteobacteria was phylum-level biomarker. The LDA results showed that the acidification group had 5 different biomarkers (LDA > 3, *P* < 0.05), of which f_Lachnospiraceae and o_Nitrospirales were the main microorganisms. The control group also had five different biomarkers (LDA > 3, *P* < 0.05), of which c_Gammaproteobacteriag and g_Photobacterium were the main microorganisms (Figures 9A,B). From the cladogram, it can be seen that the abundance of the biomarker Proteobacteria was higher at the phylum level, indicating that it played an essential role in the control group. The abundance of Lachnospiraceae in the acidification group was higher at the family level. In conclusion, acidification exposure changed the biomarker of intestines of juvenile horseshoe crab and promoted the proliferation of specific bacteria.

#### Function Prediction

**FIGURE 10:**
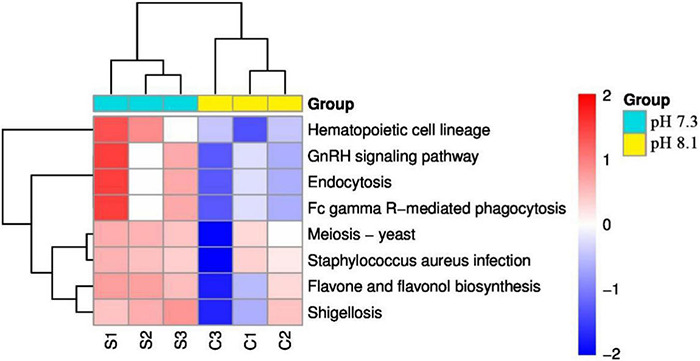
Heatmap of predicted functions of juvenile horseshoe crabs. Red indicates higher abundance; blue indicates lower abundance.

KEGG pathway analysis showed that acidification greatly alters the function of the intestinal microbiome of the juvenile horseshoe crab. “Hematopoietic cell lineage,” “Endocytosis,” and “Fc gamma R-mediated phagocytosis” pathways significantly increased in the acidification group. In addition, “*Staphylococcus aureus* infection” and “Shigellosis” pathways were also found more abundant in the acidification group (Figure 10).

## Discussion

In this experiment, 1st instar larvae were chosen because the juveniles are more sensitive to environmental changes than adults (Small et al., 2016), on the other hand, 1st instar larvae mainly inhabit the intertidal area where OA frequently occurs (Meilana et al., 2021) while large size horseshoe crabs live in the deep ocean (20 ∼ 60 m deep). In addition, materials of other life history stages are not easily obtained compared with the 1st instar larvae. Thus, 1st instars as experimental animals are appropriate. Study showed that the survival rate of 1st instar horseshoe crab larvae can reach 96% in sand sediment environment (Hong et al., 2009). In this study, acidification exposure had little effect on the survival and molting rate, indicating that the 1st instar horseshoe crab could adapt to environmental mutations through specific physiological regulation, which is similar to a previous study (Hong et al., 2009). Under normal physiological conditions, the production and neutralization of ROS in animals are in balance. However, when the organism is affected by external factors, the production rate of ROS, such as superoxide anion radicals (O•−), hydrogen peroxide radicals (H_2_O_2_), hydroxyl radicals (•OH), and peroxy radicals (ROO−), will exceed the body’s clearance rate (Halliwell and Gutteridge, 1999). Excessive ROS can induce lipid peroxidation of cell membranes and change the structure and function of cells, thus harming the health of organisms. MDA is a symbolic product of antioxidant damage (Gu et al., 2020). In this study, acidification exposure significantly increased the ROS content in juvenile horseshoe crab. MDA content of the acidification group was not significantly different from the control group on the 7th and 14th day, while it was significantly higher than the control group on the 28th day, indicating that the juvenile horseshoe crab has accumulated excessive ROS after the 28 days acidification exposure, causing oxidative damage to the juvenile horseshoe crab. In the antioxidant system, SOD is one of the most effective antioxidant enzymes in the cell. SOD catalyzes O^2–^ and H^+^ to O_2_ and H_2_O_2_, respectively; CAT further decomposes H_2_O_2_ into H_2_O. Therefore, the synergy of SOD and CAT can effectively remove ROS caused by environmental stress, protecting cells from oxidative stress and damage (Ighodaro and Akinloye, 2019). In this experiment, SOD activity in the acidification group increased significantly on the 7th day and the 14th day, and the CAT activity increased significantly on the 7th day. Muralisankar et al. (2021) showed that the CAT and SOD activities and LPO content in *Litopenaeus vannamei* significantly increased after 7 weeks of acidification exposure. Hu et al. (2015) showed that the SOD and CAT activities in the digestive glands of thick shell mussel *Mytilus coruscus* increased significantly on the 7th and 14th day of acidification exposure; Zhao et al. (2019) found that *Musculista senhousia* without a prior history of transgenerational caused oxidative stress after 6 months of OA exposure while antioxidant responses were alleviated by transgenerational acclimation, which were consistent with our results. GPX is an enzyme widely existing in the body that catalyzes the decomposition of H_2_O_2_, which can protect the structure and functional integrity of cell membranes (Ighodaro and Akinloye, 2019). GPX activity in the acidification group was significantly higher than that in the control group on the 28th day, indicating that acidification activated the antioxidant system of juvenile horseshoe crabs. GST has the dual functions of eliminating peroxides and detoxification in the body, and the increase of GST can be used as a sensitive indicator of liver damage (Cailleaud et al., 2007). In this experiment, the GST activity of the acidification group did not change significantly, indicating that the body has not started the detoxification function and did not cause liver damage to horseshoe crabs. AKP is a typical hydrolase in the innate immune system of crustaceans, and its main function is to kill extracellular invaders directly. Therefore, AKP is considered as a sensitive parameter under environmental pressure (Zhang et al., 2000; Zhao et al., 2011). In the present study, acidification exposure had little effect on the survival and molting rate but induced oxidative stress with the extension of acidification exposure, indicating that long-term acidification exposure might have a negative influence on antioxidant system of juvenile horseshoe crabs.

In the process of arthropod molting, chitinase cleaves the chitin polymer into oligomers of β-*N*-acetyl-glucosamine (NAG), which are further broken down to monomer NAGs by *N*-acetyl-β-D-glucosidase (β-NAGase) (Zou and Bonvillain, 2004). Thus, chitinase and β-NAGase are often used as markers for molting signals in crustaceans (Luo et al., 2015; Chen et al., 2019). In this experiment, the chitinase activity in the acidification group was significantly lower than that in the control group on the 7th and 28th days, and the β-NAGase activity was significantly lower than that in the control group on the 7th and 14th days. Although OA did not affect the morphological characteristics and molting rate of juvenile horseshoe crab, these two chitinolytic enzyme activities decreased during the experiment, indicating that acidification had a negative effect on the molting of juvenile horseshoe crabs because chitinase and β-NAGase are closely related to molting occurrence (Spindler-Barth et al., 1990). Walther et al. (2010) found that acidification prolonged the development time and slowed down the growth of spider crab *Hyas araneus* larvae. Long et al. (2013) showed that acidification slowed down the growth and development of juvenile *Paralithodes camtschaticus* and *Chionoecetes bairdi*, which were similar to our results.

Ecdysone is considered the most direct endocrine hormone regulating the molting of crustaceans (Wei, 2012). Some scholars believe that chitinase is the product of ecdysone regulation in crustaceans (Zou and Fingerman, 1999; Zou, 2005). Our study showed that the ecdysone content of juvenile horseshoe crab was significantly lower than that of the control group after 7 days of acidification exposure. There has been little research examining the effect of OA on hormones, Mustafa et al. (2015) believed that acidification (pH 7.5) interferes with ecdysteroid secretion for the white shrimp *L. vannamei*. However, Harrington and Hamlin (2018) found that the ecdysterone concentrations of subadult American lobsters *Homarus americanus* were not significantly altered after OA exposure (pH 7.6) for 60 days. Long et al. (2021) showed that golden king crabs *Lithodes aequispinus* at pH 7.5 were smaller than crabs at ambient (pH 8.2) after 128 days’ exposure, in terms of both carapace length and wet mass, had a smaller growth increment after molting, and had a longer intermolt period. Thus, in the present study, it is indicated that OA had an adverse effect on molting-related activities for juvenile horseshoe crabs through inhibiting the ecdysone secretion.

The structure of intestinal microflora is closely related to the health of the organism (O’Hara and Shanahan, 2006) and is affected by several factors, such as genetic, food, geography, life history, and living environment (O’Hara and Shanahan, 2006; Duan et al., 2018). The balance of the intestinal environment affects the host’s growth and development, metabolic transformation, immune regulation, and many vital physiological functions (Rawls et al., 2004; Flint et al., 2008; Butt and Volkoff, 2019). In recent years, Miao et al. (2020) first studied the effects of initial feeding and first molting on the intestinal microbes of juvenile horseshoe crabs, who found that molting had a great impact on the intestinal microbes; Wang et al. (2020) compared the differences of intestinal microbiota between *Carcinoscorpius rotundicauda* and *T. tridentatus* in Beibu Gulf. Qu et al. (2020) analyzed the composition of the intestinal microbes of the tri-spine horseshoe crab in Hong Kong. All the above showed that Proteobacteria, Bacteroides, and Firmicutes were the dominant flora of *T. tridentatus*, which were consistent with our results. Aires et al. (2018) showed that the community structure and composition of algae-related bacteria did not change significantly after 3 weeks of acidification exposure, while the intestinal microbial community of *Synisoma nadejda*, a plant-eating isopod, changed significantly. Fonseca et al. (2019) showed that Firmicutes disappeared in the intestines of sea bream after 1 month of acidification exposure, while γ-shaped bacteria (Vibrionaceae and Spartinaceae) emerged. In this study, the abundance of Bacteroides and Proteobacteria in acidification group decreased, while the abundance of Sclerotinia increased compared with the control group. It is generally believed that Bacteroidetes can promote carbohydrate fermentation, which is a group of bacteria that participate in carbohydrate, bile acid, and steroid metabolism (Zhang et al., 2014). Proteobacteria can be used as a health index of fish (Foysal et al., 2019), and increasing the number of Proteus can improve the health status of Atlantic salmon (Dehler et al., 2017), which suggests that long-term OA may affect the health status of juvenile horseshoe crabs.

According to LEfSe analysis, at the genus level, Nitrospira was the biomarker in the acidification group and Photobacterium was the biomarker in the control group. Piazzon et al. (2017) found that the genus Photobacterium was the biomarker in the control group of sea bream in the fish farm, which was similar to our results. Subsequently, the KEGG pathway showed that acidification increased the expression abundance of “Hematopoietic cell lineage,” “Endocytosis,” and “Fc gamma R-mediated phagocytosis” in 1st juvenile horseshoe crab. Interestingly, ROS increased under acidification exposure. As an invertebrate, horseshoe crab mainly relies on innate immune defense system, and its hemolymph plays an important role in cellular and humoral immunity (Iwanaga, 2002). In *Pacifastacus leniusculus*, the increase of ROS content in hematopoietic tissue can initiate hematopoiesis and release the signal of newborn blood cells (Junkunlo et al., 2016). Recent studies have shown that the content of ROS plays an important role in the hemolymph cell proliferation in horseshoe crabs (Xu et al., 2021), suggesting that acidification exposure may activate the innate immune system of juvenile horseshoe crabs by generating oxidative stress, thus forcing hemolymph cells responsible for immune functions to proliferate in order to defend against the adverse external environment. At the same time, acidification also increased the possibility of *Staphylococcus aureus* infection and Shigellosis in juvenile horseshoe crabs, all of which suggest that acidification has an adverse impact on the immune function of the juvenile horseshoe crab.

To integrate the changes of chitinolytic enzymes, ecdysone, and non-specific immune enzymes parameters associated with pH reduction, PCA was used to distinguish the combinatorial effects as a function of principal components. During the experimental period, PC1 strongly reflected the changes of all parameters, and it distinguished the OA treatments from the control treatments because most of the control treatments were put together by PC1. PC2 separated the 0 and 7th days from the 14th and 28th days, indicating an exposure period effect. Pearson correlation analysis showed that chitinase was significantly positively correlated with ecdysone, which was consistent with the previous data on the *Eriocheir sinensis* (Chen et al., 2016) and *Sinopotamon henanense* (Luo et al., 2015). MDA significantly negatively correlated with chitinase and ecdysone, implying that the accumulation of lipid peroxides is not conducive to the growth of juvenile horseshoe crab, which is similar to the study of Muralisankar et al. (2021).

Based on the above, acidification induced the activities of antioxidant enzymes and non-specific immune enzymes, i.e., SOD, CAT, GPX, and AKP. MDA content were significantly higher than that of the control group on the 28th day, suggesting that acidification caused oxidative stress to juvenile horseshoe crabs. In addition, since chitinase, β-NAGase, and ecdysone are closely related to molting occurrence, acidification exposure inhibited the activities of chitinase and β-NAGase, and interfered with ecdysone expression at the 7th day, suggesting that acidification may have a negative effect on molting occurrence. However, because the molting interval of juvenile horseshoe crabs lasts for a long time, it is necessary to further investigate whether acidification affects the molting occurrence under the constant OA. In addition, acidification exposure changed the biomarker of intestinal microorganisms of juvenile horseshoe crab, and increased the possibility of *Staphylococcus aureus* infection and Shigellosis in juvenile horseshoe crabs at the end of the experiment, suggesting that OA negatively affected the immune function of juvenile horseshoe crabs.

## Conclusion

Our results indicate that short-term acidification exposure interfered with the activity of chitinolytic enzymes and ecdysone expression for juvenile horseshoe crabs, and long-term OA caused oxidative stress. OA had adverse effects on the immune defense function and intestinal health to juvenile horseshoe crabs. However, since the molting interval of juvenile horseshoe crabs lasts for a long time and the acidification exposure time is not very long in the present study, we cannot determine if acidification affects the molting rate. On the basis of our findings, further researches can be conducted to evaluate the longer term (e.g., for 1 year) physiological effects of OA on multiple life history stages and explore whether OA affect the hardness and calcification time of new carapace. This study provided reference for physiological adaptation mechanism of juvenile horseshoe crab and accumulated basic data for the assessment of the impact of OA on the marine ecosystem.

## Data Availability Statement

The datasets presented in this study can be found in online repositories. The names of the repository/repositories and accession number(s) can be found below: SRA database and PRJNA779855. The link is [https://www.ncbi.nlm.nih.gov/sra/PRJNA779855].

## Ethics Statement

All applicable international, national, and/or institutional guidelines for the care and use of animals were followed. The number of collected animals in our study was as low as possible, and the manipulation was fast and painless.

## Author Contributions

XL contributed to conception, design, investigation, and writing—original draft of the study. JL, KX, and CZ organized the database. JF performed the formal analysis. JS, ZT, and QZ contributed to the methodology. MH contributed to conception, project administration, and writing—review and editing. YW contributed to resources, supervision, and writing—review and editing. All authors contributed to the article and approved the submitted version.

## Conflict of Interest

JS is employed by Tianjin Era Biology Technology Co., Ltd. The remaining authors declare that the research was conducted in the absence of any commercial or financial relationships that could be construed as a potential conflict of interest.

## Publisher’s Note

All claims expressed in this article are solely those of the authors and do not necessarily represent those of their affiliated organizations, or those of the publisher, the editors and the reviewers. Any product that may be evaluated in this article, or claim that may be made by its manufacturer, is not guaranteed or endorsed by the publisher.
